# Development and structural characterization of an engineered multi-copper oxidase reporter of protein–protein interactions

**DOI:** 10.1074/jbc.RA118.007141

**Published:** 2019-02-15

**Authors:** Barindra Sana, Sharon M. Q. Chee, Jantana Wongsantichon, Sarada Raghavan, Robert C. Robinson, Farid J. Ghadessy

**Affiliations:** From the ‡ p53 Laboratory, Agency for Science, Technology, and Research (A*STAR), 8A Biomedical Grove, Singapore 138648, Singapore,; the § Mahidol-Oxford Tropical Medicine Research Unit, Mahidol University, Bangkok 10400, Thailand, and; the ¶ Institute of Molecular and Cellular Biology, A*STAR, 61 Biopolis Drive, Singapore 138673, Singapore

**Keywords:** enzyme mechanism, enzyme mutation, enzyme oxidase, X-ray crystallography, structure-function, protein engineering, CueO, MDM2 proto-oncogene, protein-protein interaction, antibody, protein sensor, drug screening tool

## Abstract

Protein–protein interactions (PPIs) are ubiquitous in almost all biological processes and are often corrupted in diseased states. A detailed understanding of PPIs is therefore key to understanding cellular physiology and can yield attractive therapeutic targets. Here, we describe the development and structural characterization of novel *Escherichia coli* CueO multi-copper oxidase variants engineered to recapitulate protein–protein interactions with commensurate modulation of their enzymatic activities. The fully integrated single-protein sensors were developed through modular grafting of ligand-specific peptides into a highly compliant and flexible methionine-rich loop of CueO. Sensitive detection of diverse ligand classes exemplified by antibodies, an E3 ligase, MDM2 proto-oncogene (MDM2), and protease (SplB from *Staphylococcus aureus*) was achieved in a simple mix and measure homogeneous format with visually observable colorimetric readouts. Therapeutic antagonism of MDM2 by small molecules and peptides in clinical development for treatment of cancer patients was assayed using the MDM2-binding CueO enzyme. Structural characterization of the free and MDM2-bound CueO variant provided functional insight into signal-transducing mechanisms of the engineered enzymes and highlighted the robustness of CueO as a stable and compliant scaffold for multiple applications.

## Introduction

Our present understanding of modular architectures evident in diverse classes of proteins has enabled design of engineered proteins with novel sensing properties. Whereas functional outcomes can be impacted by lesser understood, and often interrelated phenomena such as protein stability, allostery, and epistasis, several enzymes have been modified to integrate analyte detection and signal generation components within the same host protein. A common engineering approach entails insertional mutagenesis, whereby xeno-peptides/proteins are incorporated into host proteins to generate hybrid entities with novel sensing functions (1). Insertion of a protease substrate peptide into enzyme scaffolds such as β-galactosidase can convert these to protease sensors, with target protease engagement typically destabilizing the enzyme and resulting in measurable loss of activity (2, 3). A further iteration comprises a host reporter protein inhibited in *cis* by fusion to an inhibitory domain. Proteolytic cleavage releases the inhibitory domain, resulting in measurable signal turn-on as described using β-lactamase, RNase A, p53, NIa, and NS3 reporter proteins (4–7).

Incorporation of antigenic peptides can also discern binding by specific antibodies. Using this approach, engineered β-galactosidase, alkaline phosphatase, and β-lactamase variants have been described with activities modulated by antibody binding (8–10). A reciprocal approach utilizing GFP–antibody hybrids further enables intracellular detection of antigenic peptides (11). In this case, fluorescence readout of the hybrid protein is enhanced by peptide binding.

Exposed loop regions gleaned from *a priori* structural data are typically exploited as peptide insertion sites. Random insertion coupled to selection has also been described for β-lactamase variants that bind and sense anti-prostate-specific antigen antibodies (8). Larger protein domains have been inserted into the β-lactamase, maltose-binding protein, GFP, calmodulin, and dihydrofolate reductase hosts via rational or random approaches to yield allosteric biosensing chimeras recognizing small-molecule and metal analytes (12–19).

Desirable properties of an ideal host protein are known structure, insertional tolerance proximal to active site, simple enzymatic readout, elevated thermostability, and ease of recombinant production. The *Escherichia coli* multi-copper oxidase CueO displays many of these criteria but has not been validated as a host scaffold. CueO plays an important role in copper homeostasis by oxidation of toxic cuprous ions to cupric ions (20–23). As with all multi-copper oxidases, it contains four copper atoms distributed within one type 1 (T1)^2^ copper site and a trinuclear cluster comprising the T2 and T3 copper sites. A further Cu(I)-binding site, termed the substrate copper (sCu) site or T4 lies proximal to T1, and its occupancy is linked to oxidation of proximally bound polyphenols, metal ions, and aromatic polyamines (24). A four-electron transfer between these sites couples substrate oxidation to reduction of dioxygen bound to the trinuclear site, with commensurate production of water. A distinguishing feature of CueO is a partially structured 45-amino acid segment (residues 356–404) capping the entrance to the T1/sCu copper-binding sites (25). Mutagenesis studies indicate this methionine-rich segment (MRS) to be important for both Cu(I) binding and regulation of substrate specificity (26). Notably, complete deletion of the MRS (with replacement by a minimal dipeptide linker) does not abrogate function, instead leading to emergence of altered/novel substrate specificities (27). Both the inherent plasticity and substrate-binding site proximity of the MRS make CueO an attractive host for comprehensive engineering.

The goal of the current study was to engineer the highly compliant MRS such that CueO activity would be modulated by engagement of a partner protein with a scaffolded peptide. We first inserted peptide motifs derived from p53 that bind the N-terminal domain of the E3 ligase MDM2, a key negative regulator of the p53 tumor suppressor and therapeutic target (28–34). MDM2 engagement with the scaffolded peptides resulted in an increase in enzyme activity that could be abrogated by small-molecule and peptidic MDM2 inhibitors. Insertion of antigenic peptides resulted in an antibody-dependent abrogation of enzymatic activity. To help rationalize these opposing analyte-dependent phenotypes, we solved the structures of free and MDM2 (residues 6–125)-bound CueO. Our results validate CueO as robust host protein for use in biosensing and drug-screening applications.

## Results

### Mutational tolerance of CueO

**Figure 1.**
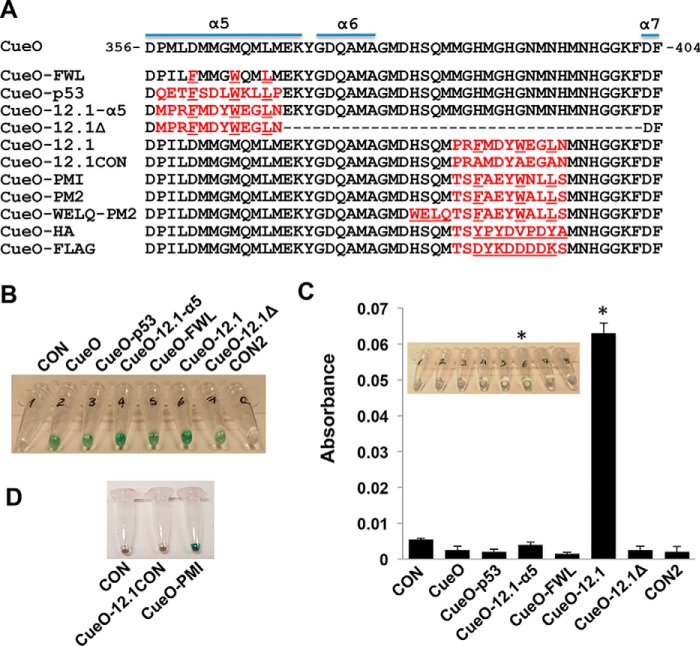
**Insertional mutagenesis into the CueO multi-copper oxidase.** *A*, CueO MRS sequence with secondary structure elements highlighted. Shown *below* are design iterations evaluated in this study. Inserted or mutated residues are depicted in *red*. The FWL motif, WELQ protease recognition sequence, and HA and FLAG epitopes are *underlined. B*, activity measurement of engineered CueO constructs by simple colorimetric readout. *CON*, no enzyme expression cassette added to the IVT reaction. *CON2*, no IVT reaction added. *C*, the indicated CueO variants were incubated with immobilized MDM2, and bead-bound activity was measured by incubation with ABTS substrate. Both absorbance reading (*A*_530_) and visual readout (*inset*) are shown. *D*, visual readout indicating interaction of CueO-PMI with MDM2 measured by pulldown assay.

A panel of CueO variants was generated with differing modifications in the MRS (Fig. 1*A*). These included insertion of the parental MDM2-binding peptide sequence present in the N-terminal domain of p53 along with a higher-affinity derivative (peptide 12.1) (35) into the MRS α5 helix to generate CueO-p53 and CueO-12.1-α5, respectively. C-terminal residues in the MRS were further deleted in the latter construct to generate CueO-12.1Δ. These MDM2-binding peptides comprise obligate Phe, Trp, and Leu residues (underlined) essential for high affinity binding (36). A further triple point mutant (CueO-FWL) was constructed with these residues introduced into the α5 helix in the same register observed for the MDM2-binding peptides. Peptide 12.1 and a control peptide with the Phe, Trp, and Leu residues mutated to alanine were also inserted in the intrinsically disordered region of the MRS (linking helices α6 and α7) to yield variants CueO-12.1 and CueO-12.1CON, respectively. Mutational tolerance was assayed by *in vitro* translation coupled to a rapid colorimetric readout of oxidase activity using 2,2′-azino-bis(3-ethylbenzothiazoline-6-sulfonic acid) (ABTS) substrate. All variants displayed readily observable enzymatic activity, highlighting the robustness of the CueO scaffold (Fig. 1*B*).

### Interaction of CueO variants with MDM2

We next assayed the engineered CueO panel by pulldown assay, using MDM2 as bait and measuring residual bead-bound enzyme activity. The results showed interaction with MDM2 only for the CueO-12.1 variant, where the 12.1 peptide replaced residues 384–394 in the intrinsically disordered region of the MRS (Fig. 1*C*). Having delineated the MRS subregion suitable for introduction of peptide sequences, we produced two additional constructs (CueO-PMI and CueO-PM2) (Fig. 1*A*) using previously identified higher-affinity MDM2 binding peptides (32, 37). The affinity of these variants for recombinant MDM2 (residues 6–125 comprising the p53 binding domain) was first measured by fluorescence polarization. Both variants showed high affinity to MDM2 (apparent *K_d_* 28 ± 1 and 25 ± 1.5 nm, respectively), comparable with affinities of their unmodified linear and stapled versions (Table 1) (Fig. S1). Binding of the higher-affinity CueO-PMI to full-length MDM2 was also clearly observed by visual readout in the pulldown assay (Fig. 1*D*).

**Table 1:** **Apparent binding affinities of CueO and indicated variants for MDM2(6–125)** Values represent an average of three independent fluorescence polarization experiments ± S.D.

Construct	Affinity	Free peptide	Stapled peptide
	*nm*		
CueO	44,280 ± 12,690		
CueO-12.1	722 ± 123	240 ± 54*^a^*	
CueO-12.1CON	No binding		
CueO-PMI	28 ± 1	47 ± 7*^b^*	87*^b^*
CueO-PM2	25 ± 2	28 ± 1*^b^*	34 ± 2*^b^*

*^a^* Measured by ITC (54).

*^b^* Measured by FP (37).

### Assaying MDM2 inhibition by small-molecule/peptide antagonists using CueO-PM2

**Figure 2.**
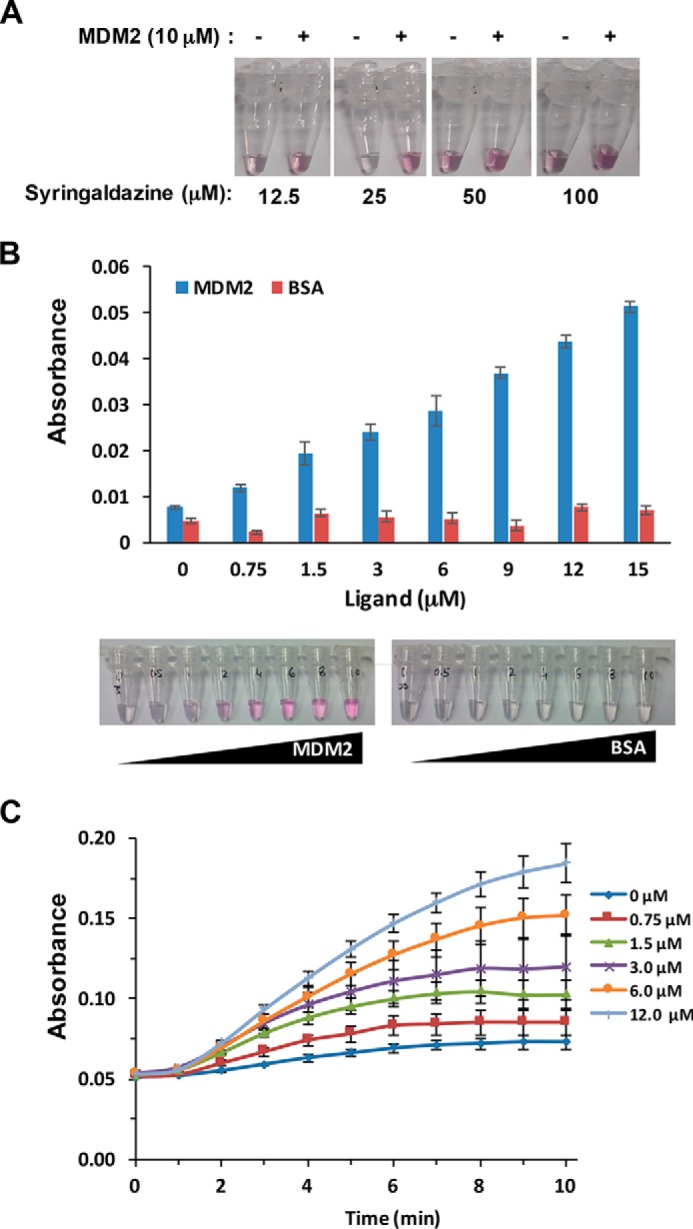
**Detection of MDM2 by CueO-PM2 in a homogeneous assay.** *A*, MDM2(6–125) (10 μm) was incubated with CueO-PM2 (16 °C for 1 h), and enzymatic activity was observed visually at the indicated concentrations of syringaldazine. *B*, MDM2(6–125) at the indicated concentrations was incubated with CueO-PM2 (16 °C for 1 h), and oxidase activity was measured using syringaldazine substrate (25 μm). Both absorbance readings at *A*_530_ (*top*) and visual readouts (*bottom*) are shown. Control experiments utilized equivalent concentrations of BSA. *C*, time course showing signal over time (minutes) as a function of the indicated MDM2 concentrations. Values represent the average of three independent experiments ± S.D.

**Figure 3.**
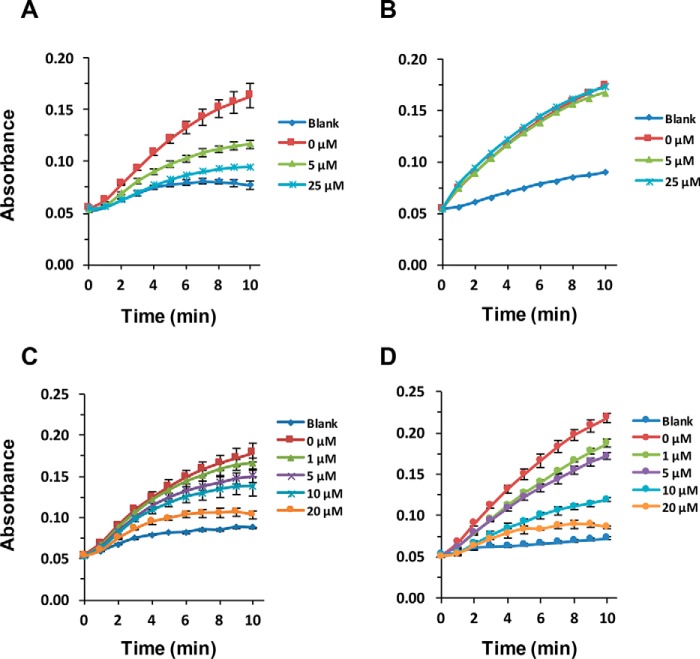
**Detection of pharmaceutical MDM2 antagonism.** *A*, CueO-PM2 (1.5 μm) was incubated with 7.5 μm MDM2(6–125) and the indicated concentrations of PM2 stapled peptide. After incubation (16 °C, 1 h) syringaldazine substrate was added, and enzyme activity was measured over 10 min. *B*, as in *A*, with PM2 control stapled peptide. *C*, as in A, with small-molecule MDM2 inhibitor RG7112. *D*, as in *A*, with AMG232 small-molecule inhibitor. Values represent the average of three independent experiments ± S.D.

Enzymatic activity of CueO-PM2 after incubation with MDM2 (10 μm) was next assayed at varying concentrations of syringaldazine substrate (12.5–100 μm). Clear MDM2-dependent potentiation of CueO-PM2 activity was observed, with maximal signal differentiation (with or without MDM2) observed visually using 25 μm syringaldazine (Fig. 2*A*). This concentration was used for all subsequent experiments. Titration of MDM2 indicated dose-responsive enhancement of CueO-PM2 activity, with spectroscopic and visual limits of detection around 750 nm and 3 μm, respectively (Fig. 2, *B* and *C*). No activation was seen using BSA control at the same concentrations. To further demonstrate specificity, the assay was repeated in the presence of competitive inhibitors. PM2 stapled peptide (37) competed with CueO-PM2 for MDM2 binding (Fig. 3*A*), resulting in signal attenuation that was not observed for a control stapled peptide (PM2-CON) (Fig. 3*B*). The small-molecule MDM2 inhibitors RG7112 and AMG232 (38, 39), both presently in clinical trials for treatment of leukemia, also showed dose-responsive attenuation of signal (Fig. 3, *C* and *D*).

### Detection of antibodies using engineering CueO variants

**Figure 4.**
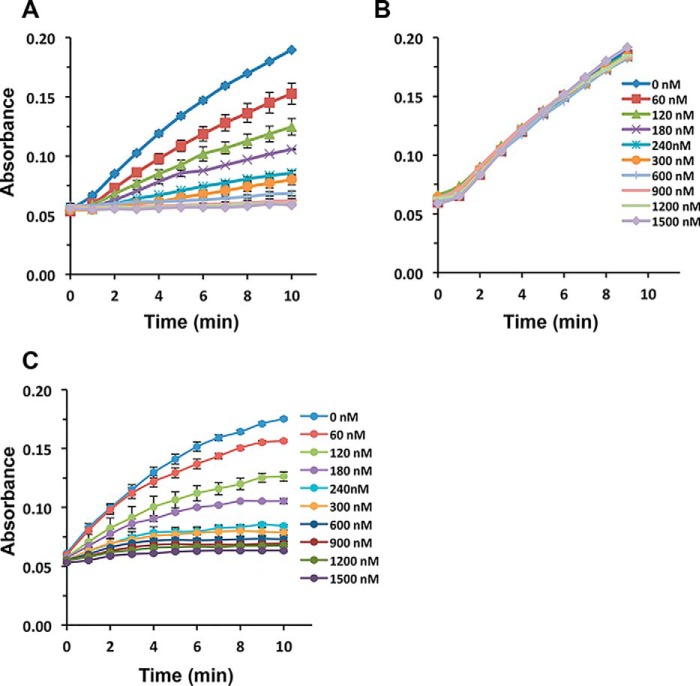
**Detection of antibodies by engineered CueO enzymes.** *A*, CueO-HA (1.5 μm) was incubated with the indicated concentrations of anti-HA antibody at 16 °C. After 1 h, syringaldazine substrate was added and enzyme activity was measured over 10 min at *A*_530_. *B*, as in *A*, incubated with anti-Myc antibody. *C*, CueO-FLAG (1.5 μm) was incubated with the indicated concentrations of anti-FLAG antibody at 16 °C. After 1 h, syringaldazine substrate was added, and enzyme activity was measured over 10 min at *A*_530_.

Antibodies commonly recognize short linear peptide sequences in target proteins. We therefore focused on recapitulating antibody–peptide interactions in the context of a CueO-scaffolded antigenic peptide. The human influenza hemagglutinin (HA) epitope was incorporated into the MRS and CueO-HA activity assayed in the presence of anti-HA or nonspecific (anti-Myc) antibody (Fig. 4, *A* and *B*). Only specific antibody resulted in clear dose-responsive reduction of enzyme activity, with an ∼60 nm limit of detection. Incorporation of the FLAG epitope (CueO-FLAG) led to detection of anti-FLAG antibody (Fig. 4*C*), further highlighting modularity of the CueO scaffold.

### Enzymatic activity and kinetics of engineered CueO variants

**Figure 5.**
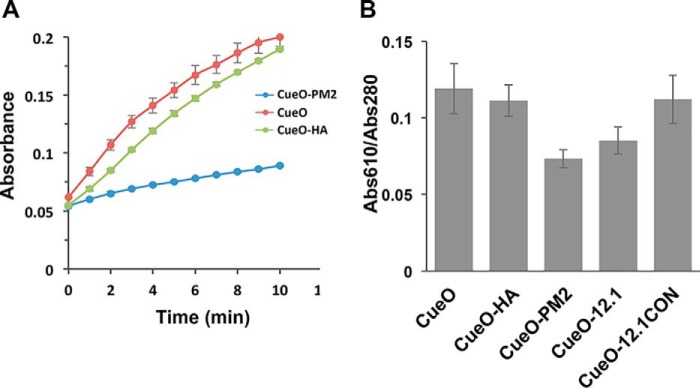
*A*, activity of CueO and derived sensors was measured over 10 min in the absence of analyte. *B*, measurement of Cu occupancy at T1 sites for CueO and the indicated sensor constructs (*n* = 3 ± S.D.).

To understand the opposing signal readouts generated upon MDM2 and antibody binding, we compared enzymatic activities of CueO, CueO-PM2, and CueO-HA in the absence of protein analytes. Whereas the activities of CueO and CueO-HA were similar, CueO-PM2 showed ∼2-fold reduced activity (Fig. 5*A*), indicating auto-inhibition by the PM2 peptide that is relieved upon MDM2 interaction. Kinetics data indicated that insertions into the MRS primarily affected *k*_cat_ for syringaldazine oxidation, with no marked influence on affinity (Table 2). This was most pronounced for the 12.1 and PM2 peptide insertions, reducing *k*_cat_ ∼2.2- and 2.8-fold, respectively. In the case of CueO-PM2, co-incubation with MDM2 resulted in ∼1.6-fold increased *k*_cat_ (and no change in *K_m_*). Notably, the *k*_cat_ value for CueO-12.1 was ∼1.6-fold lower than the control CueO-12.1CON, implicating one or more of the Phe, Trp, and Leu residues present in 12.1 (and also PMI/PM2) as being responsible for the inhibitory phenotype. No marked difference in affinity for syringaldazine was observed between these two proteins. Perturbation of copper (Cu) binding at the T1 site could account for the observed *k*_cat_ deficits in the absence of analyte binding. In agreement, Cu occupancy at the T1 site as measured by absorbance at 610 nm (40) was reduced in the CueO-PM2/12.1 constructs compared with CueO, CueO-HA, and the control CueO-12.1CON (Fig. 5*B*).

**Table 2:** **Kinetic parameters of engineered CueO variants** Values represent the average of three independent experiments ± S.D. The M358I mutation is present in all variants developed in this study. It is reverted to Met in the CueO-WT construct.

Construct	*V*_max_	*K_m_*	*k*_cat_	*k*_cat_/*K_m_*	Relative efficiency
	μ*m min*^−*1*^	μ*m*	*min*^−*1*^	*min*^−*1*^ μ*m*^−*1*^	%
CueO-WT	14.37 ± 0.78	293.18 ± 23.08	151.3 ± 8.17	0.52	98
CueO (M358I)	12.78 ± 1.08	256.09 ± 9.59	134.5 ± 11.42	0.53	100
CueO-HA	8.51 ± 0.86	286.06 ± 22.89	89.6 ± 9.05	0.31	58.49
CueO-HA + anti-HA	2.68 ± 0.19	175.37 ± 11.65	28.18 ± 2.05	0.16	30.19
CueO-PM2	4.63 ± 0.16	284.77 ± 25.50	48.69 ± 1.73	0.17	32.07
CueO-PM2 + MDM2	7.29 ± 0.64	251.12 ± 17.48	76.69 ± 6.72	0.31	58.49
CueO-12.1	5.86 ± 0.35	290.65 ± 44.33	61.73 ± 3.68	0.21	39.62
CueO12.1CON	9.18 ± 1.36	234.51 ± 41.99	96.67 ± 14.29	0.41	77.36

### Structural characterization of CueO variants

**Figure 6.**
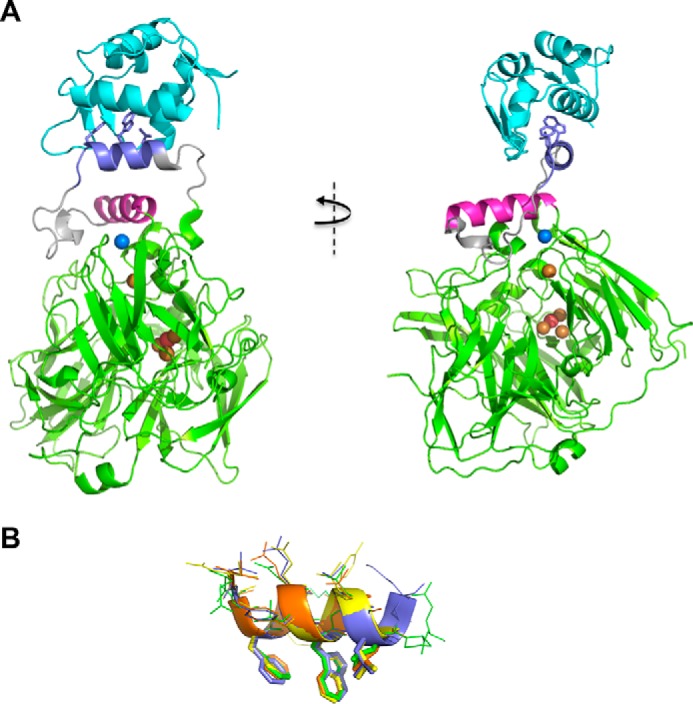
**Crystal structure of MDM2(6–125)-bound CueO-PM2.** *A*, CueO-PM2 bound to MDM2(6–125). CueO-PM2 is depicted in *green*, with the MRS and α5 helix shown in *gray* and *magenta*, respectively. Side chains of the Phe, Trp, and Leu residues of the PM2 peptide insert (*slate*) are depicted in *stick form*. MDM2(6–125) is colored *cyan*. The labile copper (*blue sphere*) along with the T1-T3 coppers (*gold spheres*) have been superimposed from the structure of copper-bound CueO (PDB code 1N68) (24). *B*, structural overlay of the CueO-scaffolded PM2 α helix (*slate*), MO6 stapled peptide (*yellow*), PMI peptide (*orange*), and parental p53 peptide (*green*). The side chains of the Phe, Trp, and Leu residues that project into bound MDM2 are depicted as *sticks*. Adapted from the PDB structures 4UMN, 3EQS, and 1YCR.

To gain further mechanistic insights, we determined crystal structures of both the free and MDM2(6–125)-bound forms of CueO-PM2 along with CueO-12.1. The asymmetric unit for the MDM2-bound form comprised a single binary complex with the scaffolded PM2 peptide adopting its prototypical α-helical conformation (Fig. 6*A*). The three key signature residues (Phe, Trp, and Leu) projecting from the outer face of the helix are accommodated by discrete pockets in a prolonged hydrophobic cleft of MDM2. Comparison with structures of unscaffolded PMI, a related stapled derivate (MO6, differing by one amino acid), and the parental p53 peptide bound to MDM2 (32, 36, 41) shows highly similar side chain conformations of these residues (Fig. 6*B*). The adjacent MRS residues Met-396 and Asn-397 extend the PM2 α-helix to more fully occupy the MDM2 binding groove. The PM2 conformation is further stabilized by hydrophobic interactions between residues on its inner face (Leu-393 and Tyr-390) and Ile-358 and Met-361 on the α5 helix. In addition to a polar contact between side chains of Lys-94 of MDM2 and Gln-365 in the α5 helix, these interactions likely drive ordering of the MRS linker residues abutting PM2 that are highly mobile in native CueO (25). The MDM2-bound PM2 helix is positioned well away from the sCu and T1 copper-binding sites, permitting unhindered access to Cu and syringaldazine substrates and resulting in the observed MDM2-dependent activity gains.

**Figure 7.**
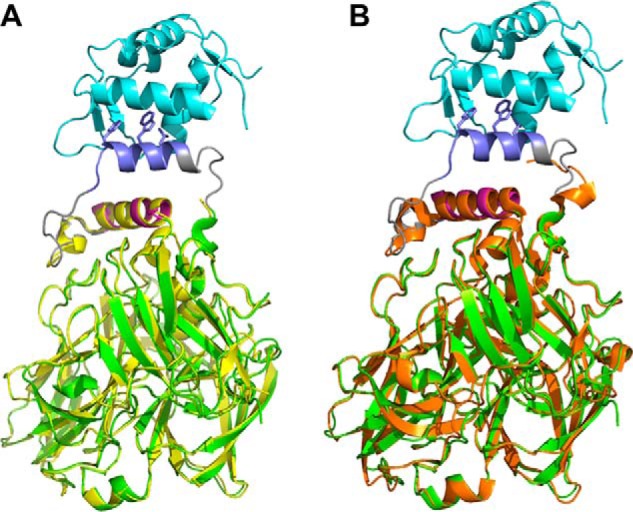
*A*, structural overlay of CueO-PM2 (*yellow*) and MDM2-CueO-PM2 complex (depicted in the *same colors* as in Fig. 6*A*). *B*, overlay of CueO depicted in *orange* (PDB code 3NSF) (26) and MDM2-bound CueO-PM2 complex.

**Figure 8.**
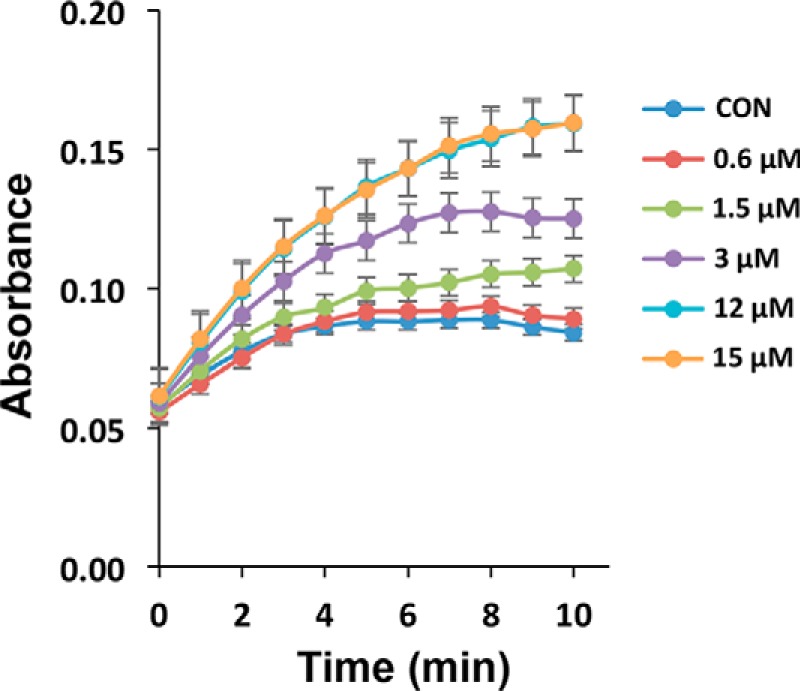
**Detection of specific protease activity using CueO-WELQ-PM2.** SplB protease was added to CueO-WELQ-PM2 at the indicated final concentrations and oxidase activity measured after a 10-h incubation at 16 °C. *CON*, no enzyme control.

The reduced activity of CueO-PM2 compared with CueO and CueO-HA along with its impaired Cu binding (Fig. 5 and Table 2) suggests that in the absence of MDM2, the PM2 helix reorients itself to block access to the sCu and T1 binding sites, most likely by packing adjacent to the CueO α5 helix. However, in the highly similar structures of CueO-PM2 and CueO-12.1 (Cα RMSD = 0.22 Å), the MRS-PM2/12.1 regions were not resolved (Fig. 7*A* and Fig. S2). The rest of the CueO-PM2 structure showed high similarity to both the MDM2-bound form (Cα RMSD = 0.3 Å) and native CueO (Cα RMSD = 0.37 Å) (Fig. 7, *A* and *B*). As the PM2-containing MRS retains some intrinsic disorder, a transient conformation likely inhibits CueO function, with all or a subset of the Phe, Trp, and Leu residues in PM2 contributing significantly (Table 2). We reasoned that formation of this transient conformation would be disrupted by significantly increasing conformational flexibility of the scaffolded PM2 insert. The cleavage motif for the site-specific SplB protease (WELQ) (42) was therefore introduced adjacent to the PM2 peptide to generate CueO-WELQ-PM2 (Fig. 1). As predicted, incubation with SplB protease resulted in dose-dependent increases in enzyme activity with an ∼1.5 μm limit of detection (Fig. 8), highlighting further use of the CueO scaffold in protease-sensing applications.

## Discussion

We have demonstrated modular engineering of the CueO enzyme to yield sensing components with integrated detection and signal-generation functions. Identification of a highly compliant peptide insertion site facilitated specific detection of diverse proteins including antibodies, an E3 ligase, and a site-specific protease. A discrete peptide sequence is thought to make a dominant contribution in one protein of an interacting pair in >50% of globular protein–protein interactions (43). As exemplified for detection of MDM2 inhibitors, CueO-based sensing therefore has significant potential to interrogate pharmaceutical antagonism of other therapeutically relevant protein–protein interactions.

Ligand binding to the engineered enzymes predominantly impacted substrate turnover and not affinity, as observed for other sensors (44). The precise binding site(s) of syringaldazine and other organic substrates in CueO is unknown, although mechanistic considerations imply proximity to the sCu/T1 coppers. Mutations in the vicinity of the CueO sCu/T1 binding sites can impact Cu binding and enzyme redox potentials both positively and negatively (24, 40, 45). The reduced T1 copper occupancy observed in CueO-PM2 likely accounts for its reduced *k*_cat_, with this phenotype contingent on either all or a subset of the Phe/Trp/Leu residues in the PM2 insert. The structure of MDM2 bound to CueO-PM2 showed clear dislocation of the PM2 insert away from the sCu and T1 copper-binding sites, rescuing enzymatic activity. Whereas the antigenic HA peptide insert similarly repressed catalytic turnover but not substrate binding relative to endogenous CueO (Table 2), antibody binding led to further abrogation and not rescue of activity. Both this and the reciprocal phenotype seen with CueO-PM2 have been observed in other antibody sensors, with positive modulation generally correlated to smaller ratios (∼0.1–0.5) between the *k*_cat_ of the engineered and the endogenous enzyme in the absence of antibody ligand (8, 44, 46). In agreement, CueO-PM2 meets this criterion (*k*_cat_ CueO-PM2/*k*_cat_ CueO = 0.32), and MDM2 binding results in increased activity. As in the case of CueO-HA, larger ratios (>0.5) most often result in antibody-dependent repression of enzymatic activity (*k*_cat_ CueO-HA/*k*_cat_ CueO = 0.58). Further structural and kinetic analysis of peptide inserts targeting other protein ligands will yield information on the generality of the observed responses.

Whereas the engineered CueO variants show modest sensitivity, performance can likely be improved by systematic modifications addressing peptide valency and/or refinements to lengths of adjacent MRS regions (47). Appendage of further ligand-binding modules by genetic fusion or protein ligation can also impart significant gains in sensitivity (6, 48). The intrinsic thermostability of CueO (*t*_½_ = 38 min at 60 °C) (27) and evident mutational plasticity of its MRS will allow comprehensive analysis of numerous design iterations guided by the structures reported here and computational analysis (49). The development cycle could be further accelerated by high throughput analysis using synthetic droplet–based microfluidics to measure oxidase activity (50).

## Experimental procedures

### Cloning and mutagenesis

The templates and the primers used for making various constructs are shown in Table 3. The CueO gene was amplified from *E. coli* JM109 genomic DNA using the primers 1 and 2 and cloned in NdeI/BamHI double-digested pET22b vector by infusion cloning. All other constructs were derived from this construct by site-directed or insertional mutagenesis (Table 3). The conservative M358I mutation arose during cloning of CueO from *E. coli* and is present in all CueO variants analyzed. It has no significant effect on enzyme activity (Table 2).

**Table 3:** **List of primers used for making CueO constructs**

Construct	Template	Primers	
		Number	Sequence
CueO	JM109 genomic DNA	1	5′-AAGGAGATATACATATGCAACGTCGTGATTTCTTAAAATATTCC-3′
		2	5′-GCTCGAATTCGGATCCTTATACCGTAAACCCTAACATCATCCCCGT-3′
CueO-FWL	CueO	3	5′-ACCCGATACTCTTTATGATGGGGTGGCAGATGCTAATGGA-3′
		4	5′-TCCATTAGCATCTGCCACCCCATCATAAAGAGTATCGGGT-3′
CueO-12.1-α5	CueO	5	5′-ATTATTGGGAAGGCCTGAACGAGAAATATGGCGATCAGGCGA-3′
		6	5′-CCATAAAGCGCGGCATGTCCATAGAGAGTTGCAGCTTGCGT-3′
CueO-12.1Δ	CueO-12.1	7	5′-GATTTCCACCATGCCAACAAAATCAACGGTCAGGCGT-3′
		8	5′-GTTCAGGCCTTCCCAATAATCCATAAAGCGCGGCAT-3′
CueO-12.1	CueO	9	5′-TGGGAAGGTTTGAATATGAACCACGGCGGGAAGTTC-3′
		10	5′-GTAATCCATAAAGCGTGGCATCTGGCTGTGATCCATCCCGGCCAT-3′
CueO-12.1CON	CueO-12.1	11	5′-CAGCCAGATGCCGCGCGCTATGGATTATGCGGAAGGCGCGAACATGAACCACGGC-3′
		12	5′-GCCGTGGTTCATGTTCGCGCCTTCCGCATAATCCATAGCGCGCGGCATCTGGCTG-3′
CueO-PMI	CueO-12.1	13	5′-ATTGGAACCTGCTGAGCATGAACCACGGCGGGAAGTTC-3′
		14	5′-ATTCCGCAAAGCTGGTCATCTGGCTGTGATCCATCCCGGCCAT-3′
CueO-PM2	CueO-12.1	15	5′-ATTGGGCGCTGCTGAGCATGAACCACGGCGGGAAGTTC-3′
		16	5′-ATTCCGCAAAGCTGGTCATCTGGCTGTGATCCATCCCGGCCAT-5′
CueO-WELQ-PM2	CueO-PM2	17	5′-TGGGAGTTACAAACCAGCTTTGCGGAATATTGGG-3′
		18	5′-ATCCATCCCGGCCATCGC-3′
CueO-HA	CueO-PM2	19	5′-TTCCAGACTACGCTATGAACCACGGCGGGAAGT-3′
		20	5′-CATCGTATGGATAGCTGGTCATCTGGCTGTGATC-3′
CueO-FLAG	CueO-PM2	21	5′-GACGATGATAAAAGCATGAACCACGGCGGGAA-3′
		22	5′-GTCTTTGTAATCGCTGGTCATCTGGCTGTGATCCA-3′
CueO-p53	CueO-PM2	23	5′-GTGGAAATTATTACCCATGAACCACGGCGGGAAGTTCGATTTCCA-3′
		24	5′-AAATCGCTAAAGGTCTCCATCTGGCTGTGATCCATCCCGGCCAT-3′

### In vitro protein production

CueO expression plasmids were PCR-amplified using the primers 5′-CATCGGTGATGTCGGCGAT-3′ and 5′-CGGATATAGTTCCTCCTTTCAGCA-3′ to generate linear expression constructs. These were column-purified (Qiagen), and 20 ng of each was used per 10-μl *in vitro* transcription/translation (IVT) reaction (New England Biolabs) carried out for 1 h at 37 °C. Oxidase activity was measured using 1 μl of IVT-expressed protein (directly without any purification) in a 10-μl reaction further comprising 1 mm ABTS, 10 mm CuCl_2_, 37.5 mm NaOAc (pH 5.2). Postincubation (37 °C, 2 h) absorbance was measured at 420 nm.

### Pulldown assay

CueO variants and HA-tagged MDM2 were produced *in vitro* as described above. 5 μl of Dynabeads Protein G (Invitrogen) washed (twice) with 200 μl of PBS-Tween (0.1%) + BSA (0.1%) was incubated with 0.5 μg of anti-HA antibody (diluted in 3% BSA in PBS) for 30–60 min at room temperature. Unbound antibody was washed (three times) with 1 ml of PBS-Tween (0.1%) + BSA (0.1%). The beads were resuspended in 5 μl of *in vitro*-expressed MDM2-HA and incubated at room temperature for 1 h before removal of unbound MDM2-HA (1× wash in PBS-Tween). Beads were next resuspended in 10 μl of *in vitro* expressed CueO protein (or variant) and incubated 1 h at room temperature. Beads were extensively washed (twice) with 1 ml of PBS-Tween (0.1%) + BSA (0.1%) and (twice) with 1 ml of PBS. Washed beads were suspended in 30 μl of buffer comprising 42.5 mm NaOAc (pH 5.2), 1 mm ABTS, and 10 mm CuCl_2_. Reactions were incubated for 1 h (37 °C), and absorbance was measured at 420 nm.

### Expression and purification of CueO proteins

All CueO constructs were cloned as fusion proteins with C-terminal His_6_ tags. The constructs were then transformed into *Escherichia coli* BL21(DE3) (Invitrogen) competent cells and grown in lysogeny broth medium at 37 °C. At *A*_600 nm_ of 0.6, the cells were induced at 37 °C for 4 h with 1 mm isopropyl 1-thio-β-d-galactopyranoside before harvesting and lysis by sonication. The cell lysate was clarified and applied to a 5-ml HisTrap column (GE Healthcare) pre-equilibrated in binding buffer (50 mm Tris-HCl, pH 8, 500 mm NaCl, 20 mm imidazole, 1 mm DTT) and eluted off the column with elution buffer (50 mm Tris-HCl, pH 8.0, 500 mm NaCl, 500 mm imidazole, 1 mM DTT). For crystallographic studies, protein samples were exchanged into buffer A solution (20 mm Tris-HCl, pH 8, 1 mm DTT) using a HiPrep 26/10 desalting column and loaded onto an anion-exchange Resource Q 6-ml column (GE Healthcare) pre-equilibrated in buffer A. The column was then washed in 5 column volumes of buffer A, and bound protein was eluted using a linear gradient to 50% of buffer B (20 mm Tris-HCl, pH 8, 1 m NaCl, 1 mm DTT) over 20 column volumes. Protein purity was assessed by SDS-PAGE, and the proteins were concentrated to 3–4 mg/ml using an Amicon-Ultra (10,000 MWCO) concentrator. For enzyme activity assays, protein was buffer-exchanged against PBS buffer (Millipore).

### Protein expression and purification of MDM2(6–125)

MDM2 (amino acids 6–125) was cloned as a GST fusion protein, expressed, and purified using affinity chromatography and a Resource S cation-exchange column as described previously (41). The GST tag was cleaved from MDM2 protein prior to use. The MDM2(6–125) protein was concentrated to ∼3 mg/ml using an Amicon-Ultra (3000 MWCO) concentrator.

### Purification of MDM2-CueO-PM2 complex

Purified CueO-PM2 was incubated with purified MDM2 (1:2 molar ratio) at 4 °C for 3 h. The protein mixture was then filtered and resolved on a Superdex 200 16/60 size-exclusion column (GE Healthcare) in gel filtration buffer (50 mm Tris-HCl, pH 8, 150 mm NaCl, 1 mm DTT). Protein fractions were then assessed by SDS-PAGE, pooled, and concentrated using an Amicon-Ultra (10,000 MWCO) concentrator (Millipore).

### Enzyme activity and kinetics assays

CueO oxidase activity was determined in a reaction mixture containing 0.1 μm enzyme (5.6 μg/ml), 50 mm acetate buffer, pH 5.2, 1 mm ABTS, or 25 μm syringaldazine (0.5 mm stock dissolved in 96% EtOH) as substrate and 1 mm copper as cofactor. The reaction mixture was incubated at 37 °C for 5 min, and the bluish green color of oxidized ABTS or magenta color of oxidized syringaldazine was measured at 420 or 530 nm, respectively. Time course analysis was done in a 384-well plate at room temperature using 0.033 mm syringaldazine and 0.33 mm copper. The color change was measured at 530 nm for 10 min using an EnVision microplate reader (PerkinElmer Life Sciences).

The reactions for kinetics analysis comprised 0.1 μm enzyme, 50 mm acetate buffer, pH 5.2, 0.33 mm copper, and concentrations of syringaldazine ranging from 8.33 to 83.3 μm. Where required, CueO-PM2 and CueO-HA were preincubated with 10 μm MDM2 and 180 nm anti-HA antibody, respectively. The reaction mixtures were prepared in 384-well plates, and color change was monitored constantly for 2 min at 530 nm in the EnVision microplate reader at room temperature. Reaction velocities were calculated from the absorbance values at the linear reaction phase using the extinction coefficient = 65,000 m^−1^ cm^−1^, and kinetic parameters were calculated from the Lineweaver–Burk plot.

### Study of PM2–MDM2 interaction and competitive inhibition

To understand the interaction between PM2 peptide (grafted in CueO) and MDM2 (N-terminal), 1.5 μm CueO-PM2 was mixed with 0, 0.75, 1.5, 3.0, 6.0, and 12 μm MDM2. The mixtures were incubated at 16 °C for 1 h and then used to measure the oxidase activity using syringaldazine as substrate.

To study the MDM2 inhibition by the stapled peptide PM2, 1.5 μm CueO-PM2 was mixed with 0, 5, and 25 μm PM2 and 7.5 μm MDM2, and the mixtures were incubated at 16 °C for 1 h. The oxidase activity was measured at room temperature using syringaldazine as substrate, and the color change was monitored for 10 min measured at 530 nm. A blank experiment was done without any MDM2 addition, and a control experiment was done by adding equal concentrations of PM2-CON (a mutant PM2 peptide that does not bind with MDM2) in place of PM2. The competitive inhibition of PM2–MDM2 interaction by small molecules RG7112 (Roche Applied Science) and AMG232 (Amgen) was studied by a similar method. 1.5 μm CueO-PM2 was mixed with 7.5 μm MDM2 and 0, 1, 5, 10, and 20 μm RG7112 or AMG232. The mixtures were incubated at 16 °C for 1 h, before measuring the oxidase activity.

### Inhibition of CueO-HA/-FLAG by the respective antibodies

1.5 μm CueO-HA or CueO-FLAG proteins was mixed with 0, 60, 120, 180, 240, 300, 600, 900, 1200, and 1500 nm anti-HA and anti-FLAG antibodies, respectively. The mixtures were incubated at 16 °C for 1 h, and oxidase activity was measured at room temperature. A control experiment was done by using equal concentrations of nonspecific antibodies.

### Crystallization and structure determination

All crystals were grown at 16 °C using the sitting-drop vapor diffusion method. CueO-12.1 was concentrated to 38 mg/ml, CueO-PM2 was concentrated to 12 mg/ml, and the MDM2-bound CueO-PM2 complex was concentrated to ∼10 mg/ml. All samples were clarified by centrifugation before setting up the crystallization trials. CueO-12.1 crystals were obtained by mixing the protein with reservoir solution (20% (w/v) PEG 3000, 100 mm Tris-HCl, pH 7, 200 mm calcium acetate) at a ratio of 1:2. CueO-PM2 crystals were grown in reservoir solution (18% (w/v) PEG 4000, 10% (w/v) 2-propanol, 100 mm Tris-HCl, pH 8, 200 mm ammonium sulfate) at a ratio of 1:1. Crystals of MDM2-CueO-PM2 were grown by mixing the complex with the reservoir solution (4 m sodium formate) in a ratio of 1:1. X-ray diffraction data were collected at the National Synchrotron Radiation Research Center (NSRRC, Taiwan), beamline BL13B1, using a square-type CCD detector (ADSC QUANTUM 315r). Data were processed and scaled using the HKL2000 program package (HKL Research). Molecular replacement was achieved using PDB entry 4UMN as a search model for MDM2 and 1KV7 for CueO in PHASER (51). Restrained refinement with TLS was performed using REFMAC (52), and model building was carried out in COOT (53). Data collection and refinement statistics for obtained structures are shown in Table 4. Structure figures were prepared using PyMOL (Schrödinger, LLC, New York). Crystal structure coordinates have been deposited in the Protein Data Bank with PDB access codes 6IM7, 6IM8, and 6IM9.

**Table 4:** **Data collection and refinement statistics** Statistics for the highest resolution shells are shown in parentheses. *R*_work_ = Σ*_hkl_*‖*F*_obs_| − *k*|*F*_calc_‖/Σ*_hkl_*|*F*_obs_|. *R*_free_ = Σ*_hkl_*_⊂_*_T_*‖*F*_obs_| − *k*|*F*_calc_‖/Σ*_hkl_*_⊂_*_T_*|*F*_obs_| where *T* represents a test set comprising ∼5% of all reflections excluded during refinement.

	CueO-12.1	CueO-PM2	CueO-PM2/MDM2
**Data collection**			
Space group	*C*121	*P*12_1_1	*P*3_2_21
Cell dimensions			
*a*, *b*, *c* (Å)	76.11, 100.73, 59.04	50.34, 90.96, 60.43	83.64, 83.64, 240.17
α, β, γ (°)	90.00, 96.17, 90.00	90.00, 104.90, 90.00	90.00, 90.00, 120.00
Resolution (Å)	30.0–1.97 (2.00–1.97)	50.0–1.80 (1.83–1.80)	30.0–3.30 (3.38–3.30)
*R*_sym_ or *R*_merge_	0.03 (0.20)	0.07 (0.13)	0.05 (0.28)
*R*_pim_	0.02 (0.13)	0.04 (0.08)	0.02 (0.16)
*I*/σ*I*	33.9 (5.5)	39.3 (10.2)	28.1 (2.8)
*CC*1/2	(0.956)	(0.979)	(0.961)
Completeness (%)	99.9 (99.5)	97.6 (99.9)	98.7 (85.8)
Redundancy	3.7 (3.4)	3.6 (3.7)	5.1 (3.4)
Unique reflections	31,017 (1517)	47,465 (2417)	15,209 (850)
**Refinement**			
Resolution (Å)	29.4–1.97 (2.03–1.97)	35.9–1.80 (1.84–1.80)	28.92–3.30 (3.55–3.30)
No. of reflections	30,933 (2447)	47,096 (2682)	14,161 (1727)
*R*_work_	0.140 (0.143)	0.161 (0.189)	0.177 (0.249)
*R*_free_	0.184 (0.185)	0.194 (0.247)	0.249 (0.378)
Molecules per asymmetric unit	1	1	4
Residues modeled to each molecule			
A	30–42, 47–378, 403–518	29–379, 402–519	24–111
B			24–111
C			30–520
D			30–520
No. of atoms	3926	4124	4493
Protein	3573	3615	4493
Metal ion	1		
Water	352	509	
*B* factors			
Protein	26.9	25.5	70.3
Water	35.2	36.9	
Metal	22.3		
RMSDs			
Bond lengths (Å)	0.007	0.007	0.011
Bond angles (°)	0.860	0.874	1.215
Ramachandran (%)			
Favored	97.0	96.8	92.1
Allowed	3.0	3.2	7.2
Outliers	0	0	0.7

### Fluorescence anisotropy

Competitive fluorescence anisotropy assays were performed as described previously (41). CueO proteins were titrated against 150 nm MDM2(6–125) and 50 nm FITC-labeled 12.1 peptide. Anisotropy measurements were done using the Envision Multilabel Reader (PerkinElmer Life Sciences). All experiments were carried out in PBS solution containing 3% DMSO and 0.1% Tween 20 buffer. Curve fitting to determine apparent *K_d_* values was carried out using Prism version 5.0 (GraphPad Software).

## Author contributions

B. S. and F. J. G. conceptualization; B. S., S. M. Q. C., J. W., and S. R. data curation; B. S., S. M. Q. C., J. W., S. R., R. C. R., and F. J. G. formal analysis; B. S., R. C. R., and F. J. G. supervision; B. S. and F. J. G. validation; B. S., S. M. Q. C., J. W., S. R., and R. C. R. investigation; B. S., J. W., S. R., R. C. R., and F. J. G. methodology; B. S., S. M. Q. C., J. W., S. R., R. C. R., and F. J. G. writing-original draft; B. S. and S. M. Q. C. writing-review and editing.

## Supplementary Material

Supporting Information

## Footnotes

2 The abbreviations used are:

T1: T2, T3, and T4, type 1, 2, 3, and 4 copper sites, respectively
sCu: substrate copper
MRS: methionine-rich segment
ABTS: 2,2′-azino-bis(3-ethylbenzothiazoline-6-sulfonic acid)
HA: hemagglutinin
IVT: *in vitro* transcription/translation
MWCO: molecular weight cutoff.
