# Supplementary material for: Development and structural characterization of an engineered multi-copper oxidase reporter of protein–protein interactions
Source: J Biol Chem. 2019 Feb 15;294(17):7002–12. doi: 10.1074/jbc.RA118.007141 (PMC6497955; doi:10.1074/jbc.RA118.007141)
Supplement: Supporting Information [file supp_294_17_7002__index.html]

Development and structural characterization of an engineered multi-copper oxidase reporter of protein-protein interactions — Oxidase enzyme reporter of protein-protein interactions — Development and structural characterization of an engineered multi-copper oxidase reporter of protein–protein interactions — Oxidase enzyme reporter of protein–protein interactions — Supporting Information 

# Development and structural characterization of an engineered multi-copper oxidase reporter of protein–protein interactions

## Supporting Information

- Supporting Information (to be published online) - Supporting information for Sana et al 2019.
