# Supplementary material for: Development and structural characterization of an engineered multi-copper oxidase reporter of protein–protein interactions
Source: J Biol Chem. 2019 Feb 15;294(17):7002–12. doi: 10.1074/jbc.RA118.007141 (PMC6497955; doi:10.1074/jbc.RA118.007141)
Supplement: Supporting Information [file supp_RA118.007141_142539_2_supp_283222_pmljr9.pdf]

Supporting Information :

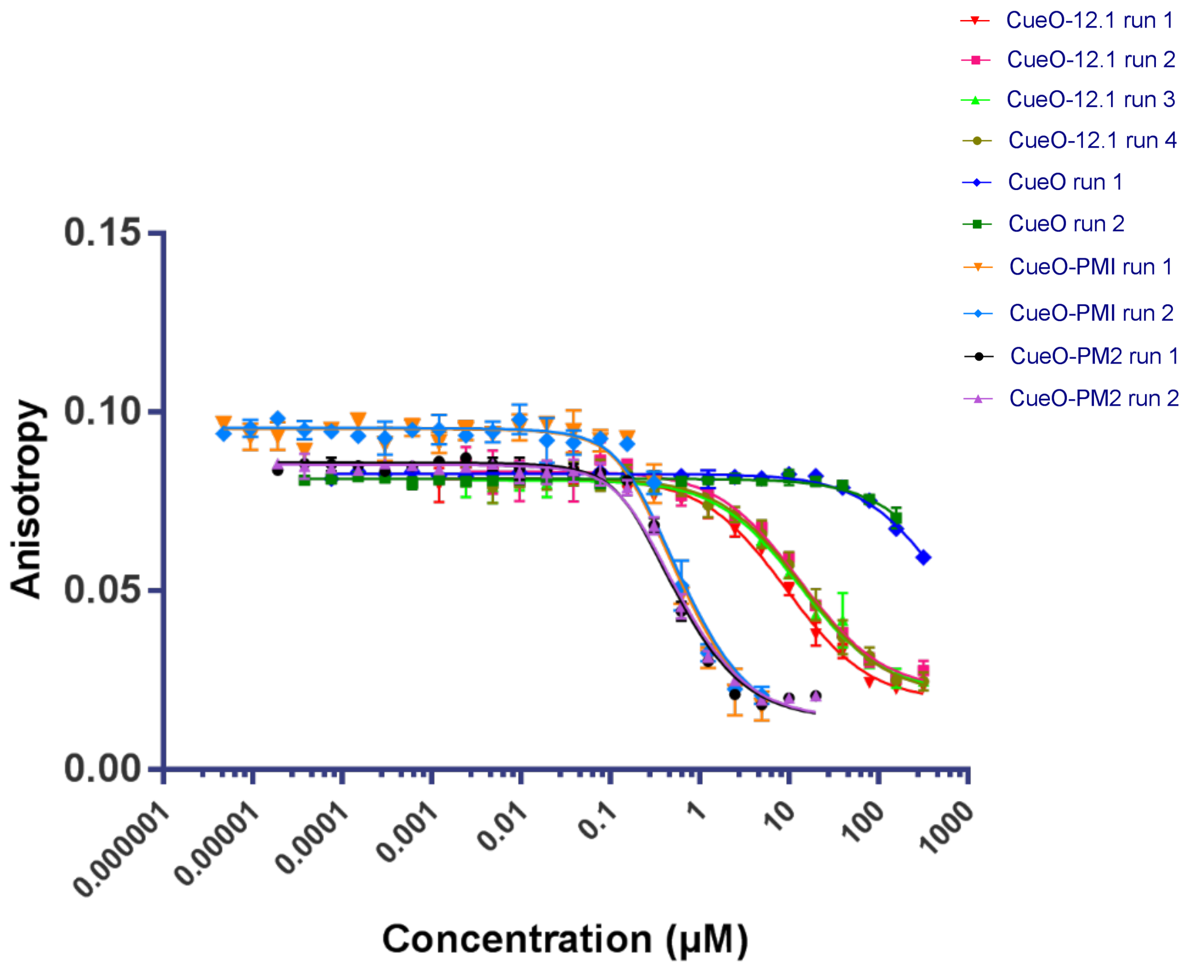

Figure S1. (A) Determination of CueO sensor affinities to MDM2 (6-125) by competitive fluorescence anisotropy. Apparent K_ds derived from titration of indicated CueO proteins with fixed concentrations of MDM2(6-125) and FAM-labelled 12.1 peptide. n= 2-4 ± SD.

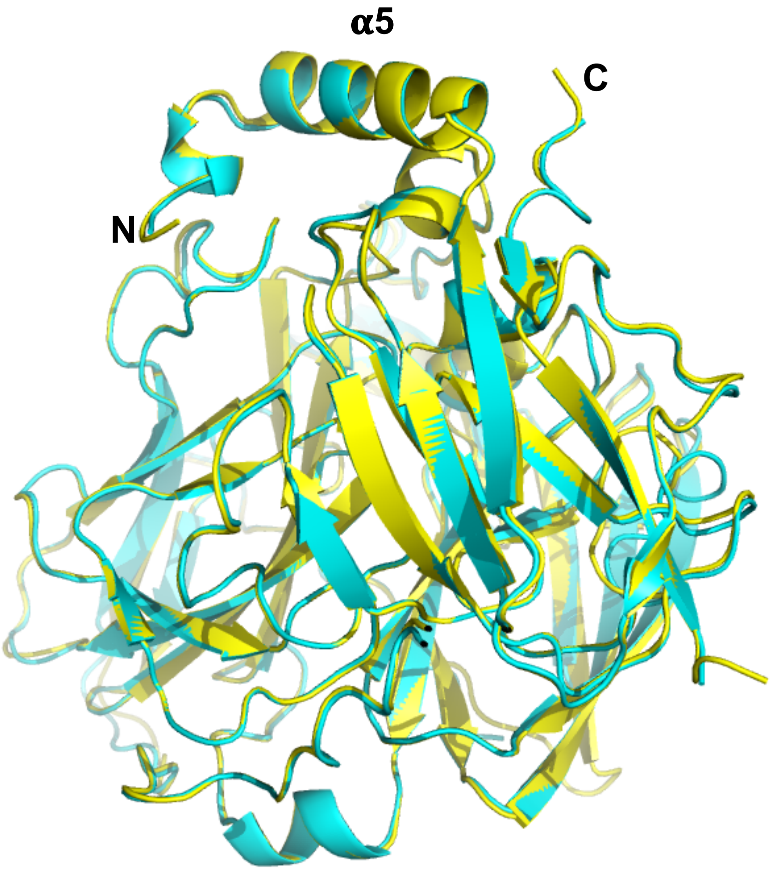

Fig S2. Structural overlay of CueO-PM2 (yellow) and CueO-12.1 (cyan). The N- and C - terminal regions flanking the unstructured region of the MRS are indicated.
